# Risk factors in critical illness myopathy during the early course of critical illness: a prospective observational study

**DOI:** 10.1186/cc9074

**Published:** 2010-06-18

**Authors:** Steffen Weber-Carstens, Maria Deja, Susanne Koch, Joachim Spranger, Florian Bubser, Klaus D Wernecke, Claudia D Spies, Simone Spuler, Didier Keh

**Affiliations:** 1 Clinic of Anesthesiology and Intensive Care Medicine, Charité University Medicine, Campus Virchow-Klinikum, Augustenburger Platz 1, 13353 Berlin, Germany; 2 Clinic of Anesthesiology and Intensive Care Medicine, Charité University Medicine, Campus Charité Mitte, Charitéplatz 1, 10117 Berlin, Germany; 3 Clinic of Endocrinology, Diabetes and Nutritional Medicine, Charité University Medicine, Campus Benjamin Franklin, Hindenburgdamm 30, 12203 Berlin, Germany; 4 Institute of Medical Biometry, Charité University Medicine, Campus Charité Mitte, Charitéplatz 1, 10117 Berlin, Germany; 5 Muscle Research Unit, Experimental and Clinical Research Center, Charité University Medicine, Campus Berlin Buch, Lindenberger Weg 80, 13125 Berlin, Germany

## Abstract

**Introduction:**

Non-excitable muscle membrane indicates critical illness myopathy (CIM) during early critical illness. We investigated predisposing risk factors for non-excitable muscle membrane at onset of critical illness.

**Methods:**

We performed sequential measurements of muscle membrane excitability after direct muscle stimulation (dmCMAP) in 40 intensive care unit (ICU) patients selected upon a simplified acute physiology (SAPS-II) score ≥ 20 on 3 successive days within 1 week after ICU admission. We then investigated predisposing risk factors, including the insulin-like growth factor (IGF)-system, inflammatory, metabolic and hemodynamic parameters, as well as suspected medical treatment prior to first occurrence of abnormal dmCMAP. Nonparametric analysis of two-factorial longitudinal data and multivariate analysis were used for statistical analysis.

**Results:**

22 patients showed abnormal muscle membrane excitability during direct muscle stimulation within 7 (5 to 9.25) days after ICU admission. Significant risk factors for the development of impaired muscle membrane excitability in univariate analysis included inflammation, disease severity, catecholamine and sedation requirements, as well as IGF binding protein-1 (IGFBP-I), but did not include either adjunctive hydrocortisone treatment in septic shock, nor administration of neuromuscular blocking agents or aminoglycosides. In multivariate Cox regression analysis, interleukin-6 remained the significant risk factor for the development of impaired muscle membrane excitability (HR 1.006, 95%-CI (1.002 to 1.011), *P *= 0.002).

**Conclusions:**

Systemic inflammation during early critical illness was found to be the main risk factor for development of CIM during early critical illness. Inflammation-induced impairment of growth-factor mediated insulin sensitivity may be involved in the development of CIM.

## Introduction

ICU-acquired muscle weakness is a serious complication of critical illness. It has been recognized as the clinical manifestation of an ICU-acquired peripheral neuromuscular pathology [1] that, with regard to muscle pathology, is characterized by atrophy of type II muscle fibres and thick filament myopathy [2].

Diagnosis of critical illness myopathy (CIM) is either based on clinical proof of muscle weakness after awakening from analgesia and sedation, measurement of short duration low amplitude muscle unit potentials, depending on voluntary muscle contraction, or histological confirmation of muscle pathology [2]. As muscle biopsies are not routinely taken at the onset of critical illness neither approach is suitable to diagnose CIM at early critical illness.

Recent studies described measurements of muscle membrane excitability after direct muscle stimulation as a valid electrophysiological marker indicating CIM in critically ill patients [2-5]. As the investigation of muscle membrane excitability is independent of voluntary muscle contraction, it enables the detection of CIM during early critical illness when clinical evaluation is generally not applicable. Attempts to determine predisposing risk factors for CIM have yielded mixed results [6-8]. Illness severity [9], duration of immobility [10], systemic inflammation, hyperglycemia [8,11,12], and the use of corticosteroids or neuromuscular blocking agents [6] are disputed risk factors. Recent data suggest a relation between growth factor-mediated dysregulation of glucose and protein metabolism due to systemic inflammation and the development of myopathy [13,14].

Measurement of muscle membrane excitability during early critical illness offers a unique opportunity to better understand and investigate early markers and potential risk factors for non-excitable muscle membrane.

The objective of this study is to investigate predisposing risk factors for the development of non-excitable muscle membrane during early critical illness, particularly considering concentration patterns of the insulin-like growth factor (IGF)-system prior to first proof of pathologically reduced muscle membrane excitability.

## Materials and methods

This study presents a subanalysis of 40 patients of a recent prospective observational study [5] that investigated the predictive value of certain electrophysiological measurements on the development of ICU-acquired weakness. Validating muscle membrane excitability at the onset of critical illness turned out to be most valuable for an early prediction of ICU-acquired weakness in immobile, sedated patients adding important information to clinical estimation of the patients' motor function upon emergence from sedation. Mechanically ventilated ICU patients on an operative ICU who featured simplified acute physiology (SAPS-II) scores of 20 or higher on three successive days within one week after ICU admission were included in the study. Sequential electrophysiological measurements including measurement of muscle membrane excitability had been performed at study enrollment and every three days until pathological findings were detected or clinical evaluation of muscle strength by Medical Research Council (MRC) score was possible.

Details of electrophysiological measurements are reported elsewhere [5], in brief we assessed the compound muscle amplitude with concentric needle electrodes after direct stimulation of the muscle. Comparable with measurement of compound muscle amplitude after nerve stimulation, this is a quantitative method and the normal data in critically ill patients are 3 mV or more [3]. Patients with non-excitable muscle membrane after direct muscle stimulation show reduced amplitudes of less than 3 mV, whereas patients with an acute neuropathy show normal amplitudes within the muscle after direct muscle stimulation. Measurement of muscle membrane excitability diagnoses myopathy but cannot exclude an additional axonal motor neuropathy. Here we focused on a risk factor analysis of non-excitable muscle membrane from the beginning of critical illness until first proof of non-excitable muscle membrane. Hence, we excluded patients being pretreated on other ICUs for more than 24 hours and only included values of risk factors of the first eight days of critical illness in the analysis. The study was approved by our local review board. Written informed consent was obtained from legal proxies.

Patients were treated following standard operating procedures of intensive care incorporating severe sepsis bundles [15]. Systemic inflammation, sepsis or severe sepsis [see Table E1 in Additional file 1] accompanied by organ dysfunction [see Table E2 in Additional file 1] was classified according to consensus conference criteria [16,17].

Inflammatory cytokines (IL-6 and IL-10), IGF-I and its binding proteins (IGFBP-I, IGFBP-III) were analysed from blood samples, drawn between days 3 and 7 as well as between days 8 and 10 after ICU admission.

Hemodynamic parameters and blood glucose levels were recorded four times daily considering least favorable values within six-hour intervals. Illness severity, SAPS-II [18], sepsis-related organ failure assessment (SOFA) [19] and other clinical data were recorded on a daily basis. Methods are further described in Additional file 1.

### Statistical analysis

Results are expressed as median and 25th/75th percentiles for continuous variables and proportions for qualitative parameters, respectively. We used nonparametric tests for statistical testing.

Changes in interesting clinical outcomes with respect to time were analyzed using nonparametric analysis of longitudinal data in a two-factorial design (1st factor: compound muscle action potential after direct muscle stimulation (dmCMAP) normal versus dmCMAP abnormal patients, 2nd factor: repetitions in time), focusing on values during the first eight days after ICU admission or within a first interval between days 3 and 7, and a second interval between days 8 and 10 after ICU admission. Therefore, we compared all time points simultaneously on the corresponding response curves [20].

In univariate and subsequently in multivariate Cox' proportional hazard regressions (stepwise backward procedure), we tested risk factors impairing muscle membrane excitability (as a dependent variable). For all parameters we included values from days of first IL-6 measurements in the analysis. Hazard ratios (HR) with their 95% confidence intervals (CI) and the corresponding *P *values were calculated for each risk factor. *P *values less than 0.05 (two-sided) were considered as statistically significant.

We evaluated the diagnostic test performance of IL-6 and SOFA to indicate the development of myopathy by receiver operating characteristics (ROC) analysis using abnormal dmCMAP amplitude less than 3 mV as electrophysiological parameter for diagnosis of myopathy and IL-6 as well as SOFA as test variables. We combined the diagnostic tests regarding sensitivity and specificity of SOFA and IL-6 to indicate myopathy with the help of the known 'believe-the-positive' rule.

All tests should be understood as constituting exploratory data analysis, such that no adjustments for multiple testing have been made. We used SPSS, Version 14 (SPSS, Inc., Chicago, IL, USA), and SAS, Version 9.1 (SAS Institute, Inc., Cary, NC, USA).

## Results

### Patient characteristics

Forty patients at the onset of critical illness were enrolled in the study. Twenty-two patients developed abnormal muscle membrane excitability in terms of reduced compound muscle action potential after direct muscle stimulation (dmCMAP abnormal) within 7 (5 to 9.25) days after admission to ICU as reported earlier [5]. Eighteen patients showed normal muscle membrane excitability (dmCMAP normal). Patients with abnormal dmCMAP revealed significant paresis (MRC 2.6 (1.84 to 3.27)) after emergence from sedation compared with patients with normal dmCMAP (MRC 4.1 (4 to 4.84); (*P *< 0.0001). ICU length of stay was significantly prolonged in dmCMAP abnormal patients (26 (18 to 38) days) compared with dmCMAP normal patients (13 (8 to 18) days; *P *< 0.0001). Patients' characteristics upon admission and within the first eight days after ICU admission are shown in Table 1.

**Table 1:** Patients' characteristics within the first week after ICU admission in patients without and with critical illness myopathy

dmCMAP		Normal	Abnormal	*P*
Number of patients	Total 40	18	22	
Age	Years	42 (24.3/58.5)	58 (42.5/68.3)	0.06^b^
Gender	male/female	12/6	15/7	1^a^
Survival	survivor/non-survivor	17/1	14/8	0.03^a^
BMI	kg/m2	24.8 (20.5/26.6)	25.4 (23.3/29.7)	0.19^b^
Reason of ICU admission	Multiple trauma. total n (%)	11 (61.1)	10 (45.5)	0.25^a^
	Pneumonia. total n (%)	3 (16.7)	7 (31.8)	
	Abdominal cancer. total n (%)	2 (11.1)	5 (22.7)	
	Others. total n (%)	2 (11.1)	0	
ICU admission	SAPS-II	31.5 (23.8/42)	41.0 (36.3/48.3)	0.02^b^
	SOFA	8.0 (5.8/10.3)	10.0 (7/12.5)	0.07^b^
	White blood cell count (1/nl)	9.6 (8.4/12.6)	8.8 (6.5/11.6)	0.26^b^
	Plasma CRP (mg/dl)	17.1 (11.03/23.9)	17.2 (10.8/26.1)	0.72^b^
	Plasma urea (mg/dl)	33 (21/53.8)	55.5 (33.8/102.5)	0.001^b^
	Plasma creatinine (mg/dl)	1.02 (0.73/1.24)	1.5 (0.92/2.6)	0.001^b^
	PaO2/FiO2	205.5 (183/247.5)	177.4 (91/256)	0.4^b^
	Plasma lactate (mmol/l)	2.15 (1.5/2.5)	3.35 (2.02/6.02)	0.02^b^
	Plasma pH	7.36 (7.3/7.47)	7.28 (7.2/7.48)	0.08^b^
Inflammation day 1-8 (cum. %)	SIRS	75.0 (52.2/100)	87.5 (71.9/100)	0.18^a^
	Severe sepsis	25.0 (0/62.5)	68.8 (9.4/87.5)	0.075^a^
	Septic shock	12.5 (0/50)	56.2 (0/87.5)	0.022^a^
Organ dysfunction day 1-8 (cum. %)	Coagulation	18.75 (0/77.7)	62.5 (9.4/100)	0.26^a^
	Lung	33.3 (18.75/75)	68.75 (25/100)	0.15^a^
	Renal	0 (0/0)	0 (0/56.3)	0.21^a^
	Liver	0 (0/31.3)	25 (0/50)	0.12^a^
	Metabolic acidosis	0 (0/18.75)	12.5 (0/50)	0.08^a^
	GCS < 13	0 (0/7.14)	6.25 (0/75)	0.2^a^
	Organ dysfunction > 2	0 (0/0)	37.5 (0/62.5)	0.003^a^
Drugs days 1 to 8 (% pat; cum dosage per pat.)	Norepinephrine (mg)	61.1; 8.9 (2.7/35.6)	91; 60.1 (27.5/84.1)	0.05^b^; 0.003^b^
	Dobutamine (mg)	27.8; 581 (259/951)	54.5; 1975 (958/4399)	0.12^b^; 0.019^b^
	NMBA (mg)	55.6; 10 (9.3/20)	63.6; 27.5 (17.5/45)	0.75^b^; 0.016^b^
	Aminoglycosides (mg)	16.7; 1440 (1260/1440)	27.3; 420 (320/620)	0.48^b^; 0.024^b^
	Hydrocortisone (mg)	16.7; 719 (501/719)	36.4; 836 (598/963)	0.29^b^; 1.0^b^
	Carbohydrates (kcal/kg)	94.4; 64.7 (29.2/103.2)	95.5; 59.7 (50.6/83.4)	1.0^b^; 0.95^b^
	Insulin (IU)	100; 237.8 (165/370)	95.5; 331.2 (155/590)	1.0^b^; 0.2^b^
	Fentanyl (mg)	94.4; 18.4 (7/26.5)	95.5; 36 (19.8/69.5)	1.0^b^; 0.006^b^
	Midazolam (mg)	77.8; 726 (318/1292)	90.9; 1702 (810/3593)	0.38^b^; 0.05^b^

Myopathy according to direct muscle stimulated compound muscle action potential (dmCMAP) at onset of critical illness, normal (≥3 mV) and abnormal (< 3 mV); parameters are calculated upon ICU admission or within eight days after ICU admission.

BMI, body mass index; CRP, C-reactive protein; GCS, Glasgow Coma Scale; NMBA, neuromuscular blocking agent; PaO2/FiO2, index of arterial oxygenation efficiency corresponding to ratio of partial pressure of arterial O2 to the fraction of inspired O2; SAPS-II, simplified acute physiology score; SIRS, systemic inflammatory response syndrome; SOFA, sequential organ failure assessment score; (cum. %), cumulative fractions of presented parameters calculated by sum of days fulfilling criteria of a particular parameter divided by total days of ICU presence within the first eight days after ICU admission; (% patients), proportion of patients; (cum. dosage), cumulative drug dose within the first eight days after ICU admission. Qualitative data are given as proportions (%). Continuous data and cumulative fractions are given as median and 25%/75% percentile; ^a^Fisher's exact test, ^b^Mann-Whitney U rank test.

Risk factors of critical illness myopathy in dmCMAP normal and dmCMAP abnormal patients within the first week after ICU admission.

**Figure 1:**
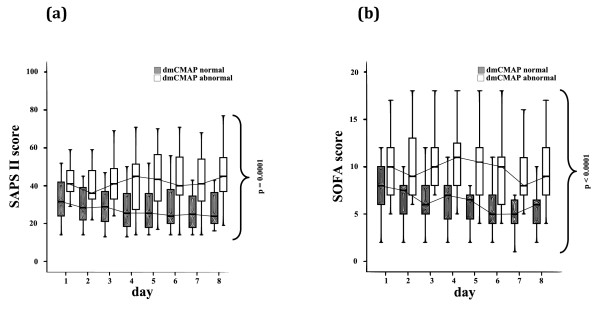
**Critical illness myopathy and disease severity scores**. Normal (≥3 mV) and abnormal (< 3 mV) direct muscle stimulation compound muscle action potentials (dmCMAP), **(a) **simplified acute physiology score-II (SAPS-II) and (B) sequential organ failure assessment (SOFA) score on the first eight days after ICU admission. Patients with impaired muscle membrane excitability had significantly higher SAPS-II and SOFA scores during the first eight days after ICU admission. Nonparametric analysis of longitudinal data in a two-factorial design (1st factor: dmCMAP normal versus dmCMAP abnormal, 2nd factor: repetitions in time. The statistical analysis was the same for Figures 1 to 6, either focusing on values from the first eight days after ICU admission (Figures 1, 2, 4 and 5) or referring to a first and second interval between days 3 and 7 after ICU admission and between days 8 and 10 after ICU admission, respectively (Figures 3 and 6).

Within the first eight days after ICU admission, patients with abnormal dmCMAP had significantly more days with systemic inflammatory response syndrome, severe sepsis, and dysfunction of two or more organs compared with patients with normal dmCMAP (Table 1 and Figure 1).

**Figure 2:**
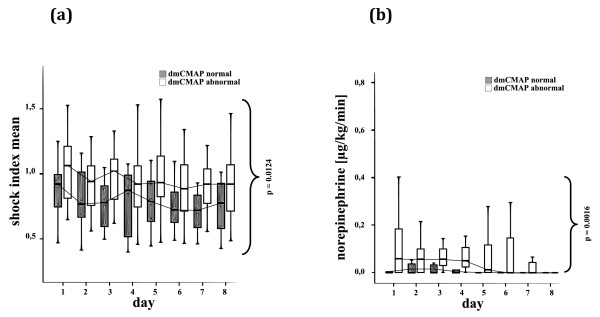
**Critical illness myopathy and hemodynamic variables**. Normal (≥3 mV) and abnormal (< 3 mV) direct muscle stimulation compound muscle action potentials (dmCMAP), **(a) **mean shock index and **(b) **daily norepinephrine dosage on the first eight days after ICU admission. Patients with impaired muscle membrane excitability had significantly higher shock indices and required significantly higher daily norepinephrine dosages during the first eight days after ICU admission.

Moreover, patients with abnormal dmCMAP received significantly higher doses of norepinephrine within the first week after ICU admission (Figure 2). Hemodynamic stability in terms of circulatory shock was significantly impaired within the first eight days compared with dmCMAP normal patients (Figure 2). There was no difference regarding frequency and cumulative dosage of adjunctive hydrocortisone therapy within the first week after ICU admission between the two groups (Table 1).

DmCMAP abnormal patients received significantly higher doses of analgesics and sedation and more neuromuscular blocking agents; however, the cumulative dosage of neuromuscular blocking agents was low within both groups (Table 1).

**Figure 3:**
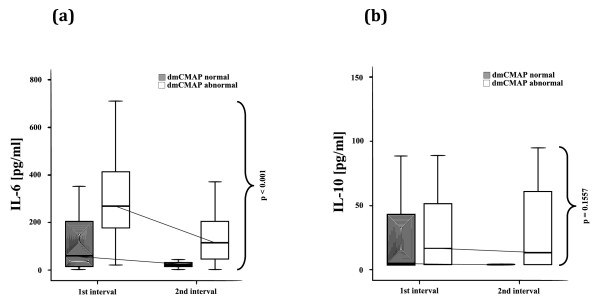
**Critical illness myopathy and systemic inflammation**. Normal (≥3 mV) and abnormal (< 3 mV) direct muscle stimulation compound muscle action potentials (dmCMAP), **(a) **IL-6 plasma levels, **(b) **IL-10 plasma levels at median day (25th/75th percentile) 5 (3 to 7) and median day 8 (6 to 10,25). Patients with impaired muscle membrane excitability had significantly higher IL-6 plasma levels but no significant differences of IL-10 plasma levels at both measurement intervals.

IL-6 plasma levels were significantly higher within the first week (day 5 (3 to 7)) in patients with abnormal dmCMAP. In the second week (day 8 (6 to 10.25)), IL-6 decreased in both groups but remained significantly higher in dmCMAP abnormal patients. There was no difference between the two groups regarding IL-10 plasma levels (Figure 3).

**Figure 4:**
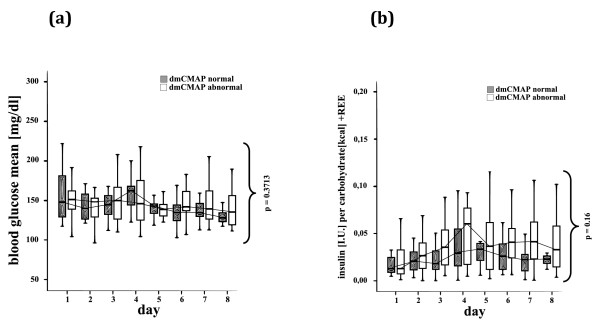
**Critical illness myopathy and glycemic control**. Normal (≥3 mV) and abnormal (< 3 mV) direct muscle stimulation compound muscle action potentials (dmCMAP), **(a) **mean blood glucose levels and **(b) **insulin in relation to daily carbohydrate intake on the first eight days after ICU admission. Mean blood glucose levels and insulin in relation to daily carbohydrate intake did not differ significantly in patients with impaired muscle membrane excitability compared with patients with normal membrane excitability.

**Figure 5:**
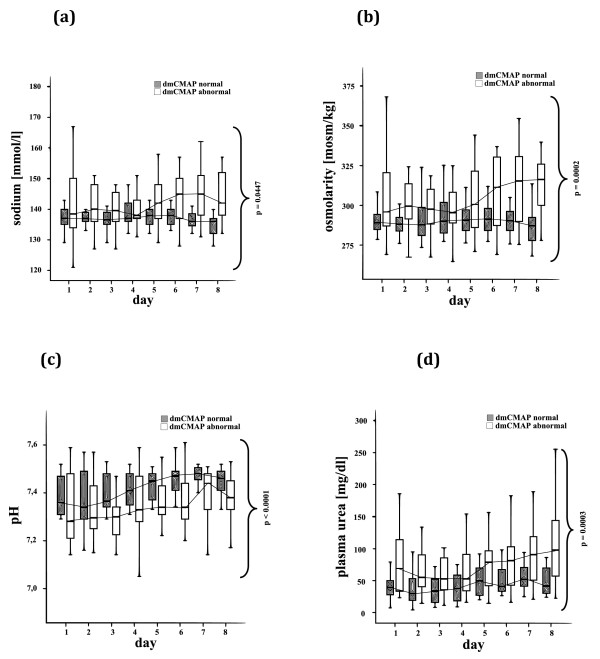
**Critical illness myopathy and plasma homeostasis**. Normal (≥3 mV) and abnormal (< 3 mV) direct muscle stimulation compound muscle action potentials (dmCMAP), **(a) **plasma sodium, **(b) **plasma osmolarity, **(c) **plasma pH and **(d) **plasma urea (multiply by factor 0.46 for blood urea nitrogen (BUN) conversion) over the first eight days after ICU admission. Patients with impaired muscle membrane excitability had significantly higher plasma sodium, plasma osmolarity, plasma pH and plasma urea during the first eight days after ICU admission.

Daily blood glucose levels (Figure 4), total carbohydrate intake, insulin requirement (Table 1), and the insulin per kcal carbohydrate intake (Figure 4) were not significantly different between the two groups within the first week. Patients with abnormal dmCMAP had a significantly higher plasma osmolarity and sodium plasma levels during the first eight days after ICU admission than dmCMAP normal patients (Figure 5).

**Figure 6:**
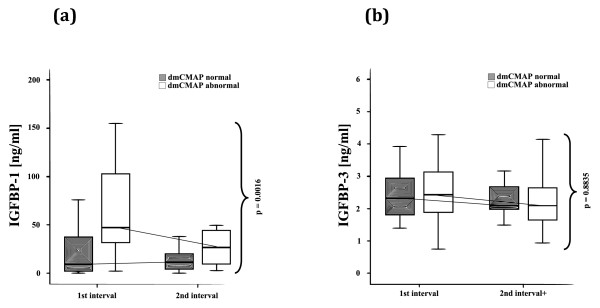
**Critical illness myopathy and insulin sensitivity**. Normal (≥3 mV) and abnormal (< 3 mV) direct muscle stimulation compound muscle action potentials (dmCMAP), **(a) **insulin-like growth factor binding protein (IGFBP)-1 plasma levels, **(b) **IGFBP-3 plasma levels at median day (25th/75th percentile) 5 (3 to 7) and median day 8 (6 to 10,25). Patients with impaired muscle membrane excitability had significantly higher IGFBP-1 plasma levels but no significant differences of IGFBP-3 plasma levels at both measurement intervals.

IGF-I was reduced in dmCMAP abnormal patients at both test intervals (87.2 ng/ml (65.9 to 119.5) versus 104.5 ng/ml (74.4 to 136.9) and 76.1 ng/ml (55.1 to 119.5) versus 87.2 ng/ml (65.8 to 122)), but differences did not reach statistical significance. Plasma levels of IGFBP-III were not different between both groups whereas IGFBP-I as a marker reflecting impaired insulin sensitivity was significantly higher in dmCMAP abnormal patients at both test intervals (Figure 6).

### Cox' regressions analysis

Separate (univariate) Cox regression analyses for risk factors impairing muscle membrane excitability are shown in Table 2. Analyses included values from day of first IL-6 measurements.

**Table 2:** Risk factors leading to impaired muscle membrane excitability

Cox' regression with time dependent covariates		Hazard ratio	95% CI		*P*
				
			LL	UL	
Univariate					
	SOFA	1.175	1.042	1.324	0.008
	SAPS-II	1.019	0.998	1.04	0.018
	Norepinephrine	1.006	1.002	1.011	0.004
	Dobutamine	1.000	1.000	1.001	0.004
	Midazolam	1.001	1.000	1.001	0.012
	Fentanyl	1.026	1.003	1.048	0.024
	HC in septic shock	1.001	0.999	1.003	0.310
	NMBA (Cisatracurium)	1.027	0.934	1.128	0.58
	Aminoglycoside (Tobramycin)	0.99	0.99	1.007	0.791
	Osmolarity	1.012	0.992	1.032	0.258
	IGFPB-1	1.012	1.004	1.020	0.003
	C-reactive protein	1.058	0.994	1.125	0.075
	IL-6	1.006	1.003	1.009	< 0.0001

Univariate Cox' proportional hazard regression with time dependent covariates for variables considered risk factors impairing muscle membrane excitability (as dependent variable) are shown. For the particular parameter repeated measures of daily cumulative dosages, daily plasma levels or daily score values until first proof of reduced muscle membrane excitability in compound muscle action potential after direct muscle stimulation (dmCMAP) abnormal patients or until ICU discharge in dmCMAP normal patients were included in the analysis. HC, adjunctive hydrocortisone treatment in septic shock; IGFBP-1, insulin-like growth factor-binding protein-1; IL-6, interleukin-6; NMBA, neuromuscular blocking agent; SOFA, sequential organ failure assessment score; SAPS-II, simplified acute physiology score. Hazard ratios (HR) with LL (lower limit) and UL (upper limit), 95% confidence intervals (95% CI) and *P *values for each variable.

In univariate analysis severity of illness, sepsis-related organ dysfunction, inflammation, catecholamine requirements, sedation requirements and an impaired insulin sensitivity turned out as significant risk factors for the development of impaired muscle membrane excitability within the early course of critical illness. An increased osmolarity, adjunctive hydrocortisone treatment in septic shock, administration of neuromuscular blocking agents and aminoglycosides were not significantly correlated with the development of impaired muscle membrane excitability.

**Figure 7:**
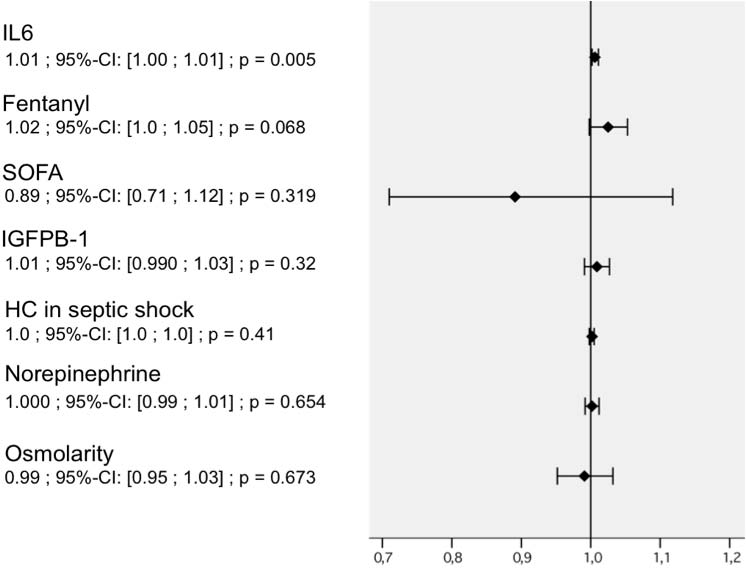
**Multivariate Cox' proportional hazard regression after backward selection for variables, that were considered as risk factors impairing muscle membrane excitability (as dependent variable)**. For the particular parameter values from the day of first blood sampling for IL-6 were included in the analysis. Hazard ratios (HR) with (95% confidence intervals (CI) and *P *values for each variable. HC, adjunctive hydrocortisone treatment in septic shock; IGFBP, insulin-like growth factor-binding protein; IL-6, interleukin-6; SOFA, sequential organ failure assessment score.

In the backward selection of multivariate Cox regression analysis (Figure 7) the extent of inflammation as reflected by IL-6 plasma levels and the fentanyl dosage remained as independent risk factors for the development of impaired muscle membrane excitability.

### Sensitivity and specificity

Sensitivity and specificity of SOFA score to predict abnormal membrane excitability was highest on day 4 at a cut-off value of 10 (sensitivity = 65% and specificity = 93.8%). The cut-off value for IL-6 predicting abnormal membrane excitability was observed at 230 pg/ml, featuring sensitivity of 71.4% and specificity of 93.3%. According to the 'believe the positive' rule applied in a combined cross tabulation of patients with SOFA scores of 10 or more at day 4 after ICU admission and/or IL-6 plasma levels of 230 pg/ml or more, we observed a sensitivity of 85.7% and a specificity of 86.7% of this combination for predicting development of abnormal dmCMAP.

## Discussion

In this observational study we investigated predisposing risk factors leading to non-excitable muscle membrane indicating CIM during early critical illness. The main finding was a significant relation between muscle membrane inexcitability, disease severity and IL-6 plasma levels.

In the absence of a reliable clinical parameter identifying patients at risk of developing CIM during early critical illness, when motor function is not assessable due to analgesia and sedation, current data on risk factors leading to CIM are derived mostly from prospective cohort studies relating data from ICU admission with patients' motor function once assessable [6]. One excellent study investigating risk factors for combined critical illness neuromyopathy [9] during early critical illness showed that illness severity, determined through acute physiology and chronic health evaluation (APACHE) III scores, predicted the later development of critical illness neuromyopathy. In our study, sequential measurements of muscle membrane excitability offers the opportunity to focus on CIM and to determine the time frame in which CIM originates during early critical illness, thereby improving stratification of predisposing risk factors for CIM.

Univariate analysis indicated illness severity, IL-6, hemodynamic impairment, decreased insulin sensitivity as well as analgesia and sedation as predisposing risk factors. However, only IL-6 and dosage of analgesia emerged as independent risk factors from multivariate analysis. Interestingly, we did not observe any significant relation between development of non-excitable muscle membrane and application of low-dose hydrocortisone, aminoglycosides or neuromuscular blocking agents, which have frequently been incriminated as being involved in the development of CIM.

Large prospective randomized studies have shown that glycemic control is associated with the development of neuromuscular dysfunction [11,12]. In our study, we did not observe a difference in blood glucose levels between dmCMAP normal and abnormal patients because we aimed for glycemic control below 150 mg/dl in all of our patients. However, despite comparable blood glucose levels between the groups, IGFBP-1 was significantly higher in abnormal dmCMAP patients. In agreement with other reports [21,22] we consider increased IGFBP-1 as a parameter indicative of impaired insulin sensitivity. Hence, our data suggest that significantly impaired insulin sensitivity during early critical illness was related to development of abnormal muscle membrane excitability.

In parallel, dmCMAP abnormal patients revealed a significant hyperosmotic state within the first days of critical illness. Hyperosmolality is related to illness severity [23] and has been described as a risk factor for critical illness neuropathy [24]. It is worth noting that the study by Garnacho-Montero and colleagues [24] did not differentiate between myopathy and neuropathy. In our study, hyperosmolality during the first days after ICU admission was significantly related to electrophysiological-proven muscle pathology. It can be speculated that this hyperosmotic state may have led to osmotic stress-induced reduction of cellular insulin sensitivity in our patients, which has been shown in adipocytes under experimental conditions [25].

However, alteration of insulin sensitivity and plasma osmolality are most likely related to systemic inflammation in our study. Our data are in agreement with the general perception that systemic inflammation and sepsis-related organ dysfunction are major triggers for the development of CIM [6,8,9,26]. Earlier experimental data inducing inflammation in rats by intravenous inoculation of endotoxin showed that IL-6 increased muscle fatigue [27] and decreased muscle contractility of the diaphragm [28]. Interestingly, recent clinical data indicate a relation between IL-6 and reduced muscle strength in elderly people [29]. Our data reveal a possible role of IL-6 in the development of non-excitable muscle membrane during early critical illness finally leading to muscle weakness.

IL-6 seems to be an important mediator leading to muscle protein breakdown [30]. One mechanism may lie in the inhibition of growth factor-mediated (e.g. IGF-I) intracellular signaling by IL-6 [13,31]. IGF-I plays a central role in glucose uptake and protein synthesis, and was shown to be downregulated in inflammation and sepsis [13]. Impairment of IGF-I may be due to inflammation-induced upregulation of high-affinity IGFBP-1 which prevents IGF-I receptor binding [32]. *In vitro *[33] and later *in vivo *[14], it has been shown that an increase of IGFBP-1 reduced the IGF-I-mediated glucose uptake and reduced protein synthesis in skeletal muscle within an experimental setting. In line with this, our data suggest that impaired growth factor-mediated intracellular signaling due to systemic inflammation may be involved in the development of CIM.

Corticosteroids are controversially discussed as aggravating factors of CIM [6,7,9,34]. It is well established that high-dose application of corticosteroids, for example in patients with chronic obstructive pulmonary disease, results in selective loss of thick myosin filaments in skeletal muscle fibers [35], a so-called steroid-induced myopathy [36].

Nevertheless, these reports refer to steroid myopathy as a result of high-dose steroid application. A link between 'low-dose hydrocortisone' treatment as adjunctive therapy during septic shock and development of CIM has been postulated [15], but never proven. Several data indicate that moderate doses of steroids do not prolong mechanical ventilation due to muscle weakness but are related to significantly more ventilator-free days and earlier spontaneous breathing capacity [37]. In an earlier study we did not observe an association between low-dose hydrocortisone application and development of paresis [5,34]. In this study we were able to show that low-dose hydrocortisone application does not provoke impaired muscle membrane excitability, suggesting that steroid involvement in CIM development is dose dependent [8].

Furthermore, dosage of analgesics and sedatives was significantly associated with the development of non-excitable muscle membrane. Interpreting higher doses of analgesics and sedatives as higher degrees of immobilization, this finding is in line with recent studies describing that immobilization aggravated neuromuscular weakness in an experimental setting [38] and that early physical mobilization resulted in a better clinical outcome of motor function [10].

For clinicians it is difficult to estimate patients at risk for the development of CIM. The APACHE-III score has been cited as being able to identify patients at risk for critical illness neuromyopathy [9]. In our study we used the SOFA score because it is widely accepted in the ICU setting, and has been validated to monitor organ dysfunction-related to sepsis [19]. Our results indicate that a SOFA score of 10 or above and/or IL-6 plasma levels of 230 pg/ml or more at the onset of critical illness disclose high-risk patients for the development of non-excitable muscle membrane.

The following limitations of this study need to be addressed. Although we observed a statistically significant effect for IL-6 as a main risk factor for non-excitable muscle membrane, it has to be stressed that the overall effect was small, which may be due to small sample size. It also needs to be mentioned that blood samples were collected at two different time points only and that the course of inflammatory parameters was not followed daily. However, this was designed as a pilot study for hypothesis generation. The clinical significance has to be addressed in further studies.

## Conclusions

Systemic inflammation during early critical illness turned out to be the main risk factor for the development of non-excitable muscle membrane indicating CIM. It may be hypothesized that inflammation-induced impairment of growth factor-mediated intracellular signaling is involved in the pathophysiology of CIM. Furthermore, adjunctive treatment with low-dose hydrocortisone during septic shock was not associated with development of CIM.

## Key messages

• Non-excitable muscle membrane indicates CIM during early critical illness.

• Inflammation, disease severity, decreased insulin sensitivity, catecholamine and sedation requirement turned out to be significantly related to the development of impaired muscle membrane excitability.

• IL-6 and dosage of analgesia emerged as independent risk factors from multivariate analysis.

• Inflammation-induced impairment of growth-factor-mediated insulin sensitivity may be involved in the development of CIM.

• In contrast to prior assumptions we could not observe any significant relation between development of CIM and application of low-dose hydrocortisone in septic shock.

## Abbreviations

APACHE: acute physiology and chronic health evaluation; CI: confidence interval; CIM: critical illness myopathy; dmCMAP: compound muscle action potential after direct muscle stimulation; HR: hazard ratio; IGF: insulin-like growth factor; IGFBP: insulin-like growth factor binding protein; IL: interleukin; MRC: Medical Research Council; ROC: receiver operating characteristic; SAPS: simplified acute physiology score; SOFA: sepsis-related organ failure assessment.

## Competing interests

The authors declare that they have no competing interests.

## Authors' contributions

SW-C conceived of the study, performed data and statistical analysis, wrote the final manuscript and is head of the project, which is funded by the Deutsche Forschungsgemeinschaft. MD and DK participated in the design of the study, in data analysis and in writing the final manuscript. SK performed the electrophysiological measurements and analysis. JS and DK performed laboratory data measurements and analysis. FB programmed the data base and participated in data collection and analysis. KW prepared the statistical part of the manuscript and performed the statistical analysis. CS critically revised the manuscript and gave final approval. SS critically revised the electrophysiological data analysis and participated in writing the paper. All authors read and approved the final manuscript.

## Supplementary Material

Additional file 1 **Further description of methods and definitions**. The additional file contains additional information on exclusion criteria, electrophysiologic measurements, general ICU care, and laboratory testing [39]. Two tables within this file explain the conditions required for defining systemic inflammatory response syndrome, sepsis, severe sepsis, or septic shock (E1) and organ dysfunction (E2). Click here for file

## References

[B1] de Jonghe B Lacherade JC Sharshar T Outin H Intensive care unit-acquired weakness: Risk factors and prevention Crit Care Med 2009 37 S309 S315 10.1097/CCM.0b013e3181b6e64c 20046115

[B2] Stevens RD Marshall SA Cornblath DR Hoke A Needham DM de Jonghe B Ali NA Sharshar T A framework for diagnosing and classifying intensive care unit-acquired weakness Crit Care Med 2009 37 S299 S308 10.1097/CCM.0b013e3181b6ef67 20046114

[B3] Trojaborg W Weimer LH Hays AP Electrophysiologic studies in critical illness associated weakness: Myopathy or neuropathy--a reappraisal Clin Neurophysiol 2001 112 1586 1593 10.1016/S1388-2457(01)00572-7 11514240

[B4] Lefaucheur JP Nordine T Rodriguez P Brochard L Origin of ICU acquired paresis determined by direct muscle stimulation J Neurol Neurosurg Psychiatry 2006 77 500 506 10.1136/jnnp.2005.070813 16306155 PMC2077517

[B5] Weber-Carstens S Koch S Spuler S Spies CD Bubser F Wernecke KD Deja M Nonexcitable muscle membrane predicts intensive care unit-acquired paresis in mechanically ventilated, sedated patients Crit Care Med 2009 37 2632 2637 10.1097/CCM.0b013e3181a92f28 19623045

[B6] De Jonghe B Sharshar T Lefaucheur JP Authier FJ Durand-Zaleski I Boussarsar M Cerf C Renaud E Mesrati F Carlet J Raphaël JC Outin H Bastuji-Garin S Groupe de Réflexion et d'Etude des Neuromyopathies en Réanimation Paresis acquired in the intensive care unit: A prospective multicenter study JAMA 2002 288 2859 2867 10.1001/jama.288.22.2859 12472328

[B7] Bolton CF Neuromuscular manifestations of critical illness Muscle Nerve 2005 32 140 163 10.1002/mus.20304 15825186

[B8] Schweickert WD Hall J Icu-Acquired weakness Chest 2007 131 1541 1549 10.1378/chest.06-2065 17494803

[B9] de Letter MA Schmitz PI Visser LH Verheul FA Schellens RL Op de Coul DA van der Meché FG Risk factors for the development of polyneuropathy and myopathy in critically ill patients Crit Care Med 2001 29 2281 2286 10.1097/00003246-200112000-00008 11801825

[B10] Schweickert WD Pohlman MC Pohlman AS Nigos C Pawlik AJ Esbrook CL Spears L Miller M Franczyk M Deprizio D Schmidt GA Bowman A Barr R McCallister KE Hall JB Kress JP Early physical and occupational therapy in mechanically ventilated, critically ill patients: A randomised controlled trial Lancet 2009 373 1874 1882 10.1016/S0140-6736(09)60658-9 19446324 PMC9906655

[B11] Van den Berghe G Wilmer A Hermans G Meersseman W Wouters PJ Milants I Van Wijngaerden E Bobbaers H Bouillon R Intensive insulin therapy in the medical ICU N Engl J Med 2006 354 449 461 10.1056/NEJMoa052521 16452557

[B12] Van den Berghe G Wouters P Weekers F Verwaest C Bruyninckx F Schetz M Vlasselaers D Ferdinande P Lauwers P Bouillon R Intensive insulin therapy in the critically ill patients N Engl J Med 2001 345 1359 1367 10.1056/NEJMoa011300 11794168

[B13] Frost RA Lang CH Alteration of somatotropic function by proinflammatory cytokines J Anim Sci 2004 82 E-Suppl E100 E109 15471789 10.2527/2004.8213_supplE100x

[B14] Lang CH Vary TC Frost RA Acute in vivo elevation of insulin-like growth factor (IGF) binding protein-1 decreases plasma free IGF-I and muscle protein synthesis Endocrinology 2003 144 3922 3933 10.1210/en.2002-0192 12933666

[B15] Dellinger RP Levy MM Carlet JM Bion J Parker MM Jaeschke R Reinhart K Angus DC Brun-Buisson C Beale R Calandra T Dhainaut JF Gerlach H Harvey M Marini JJ Marshall J Ranieri M Ramsay G Sevransky J Thompson BT Townsend S Vender JS Zimmerman JL Vincent JL International Surviving Sepsis Campaign Guidelines Committee, American Association of Critical-Care Nurses, American College of Chest Physicians, American College of Emergency Physicians, Canadian Critical Care Society, European Society of Clinical Microbiology and Infectious Diseases, European Society of Intensive Care Medicine, European Respiratory Society, International Sepsis Forum, Japanese Association for Acute Medicine, Japanese Society of Intensive Care Medicine, Society of Critical Care Medicine, Society of Hospital Medicine, Surgical Infection Society, World Federation of Societies of Intensive and Critical Care Medicine Surviving sepsis campaign: International guidelines for management of severe sepsis and septic shock: 2008 Crit Care Med 2008 36 296 327 10.1097/01.CCM.0000298158.12101.41 18158437

[B16] Levy MM Fink MP Marshall JC Abraham E Angus D Cook D Cohen J Opal SM Vincent JL Ramsay G SCCM/ESICM/ACCP/ATS/SIS 2001 SCCM/ESICM/ACCP/ATS/SIS international sepsis definitions conference Crit Care Med 2003 31 1250 1256 10.1097/01.CCM.0000050454.01978.3B 12682500

[B17] Reinhart K Brunkhorst F Bone H Gerlach H Grundling M Kreymann G Kujath P Marggraf G Mayer K Meier-Hellmann A Peckelsen C Putensen C Quintel M Ragaller M Rossaint R Stuber F Weiler N Welte T Werdan K [Diagnosis and therapy of sepsis: Guidelines of the german sepsis society inc. And the german interdisciplinary society for intensive and emergency medicine] Anaesthesist 2006 55 Suppl 1 43 56 17051663

[B18] Gall LRJ Lemeshow S Saulnier F A new simplified acute physiology score (SAPS II) based on a european/north american multicenter study [published erratum appears in JAMA 1994 may 4;271(17):1321] JAMA 1993 270 2957 2963 10.1001/jama.270.24.2957 8254858

[B19] Vincent JL Moreno R Takala J Willatts S De Mendonça A Bruining H Reinhart CK Suter PM Thijs LG The SOFA (sepsis-related organ failure assessment) score to describe organ dysfunction/failure. On behalf of the working group on sepsis-related problems of the european society of intensive care medicine Intensive Care Med 1996 22 707 710 10.1007/BF01709751 8844239

[B20] Brunner E Domhof S Langer F Nonparametric analysis of longitudinal data in factorial experiments 2002 New York: Wiley & Sons

[B21] Mesotten D Delhanty PJ Vanderhoydonc F Hardman KV Weekers F Baxter RC Van den Berghe G Regulation of insulin-like growth factor binding protein-1 during protracted critical illness J Clin Endocrinol Metab 2002 87 5516 5523 10.1210/jc.2002-020664 12466347

[B22] Borai A Livingstone C Zarif H Ferns G Serum insulin-like growth factor binding protein-1: An improvement over other simple indices of insulin sensitivity in the assessment of subjects with normal glucose tolerance Ann Clin Biochem 2009 46 109 113 10.1258/acb.2008.008160 19164337

[B23] Holtfreter B Bandt C Kuhn SO Grunwald U Lehmann C Schütt C Gründling M Serum osmolality and outcome in intensive care unit patients Acta Anaesthesiol Scand 2006 50 970 977 10.1111/j.1399-6576.2006.01096.x 16923092

[B24] Garnacho-Montero J Madrazo-Osuna J García-Garmendia JL Ortiz-Leyba C Jiménez-Jiménez FJ Barrero-Almodóvar A Garnacho-Montero MC Moyano-Del-Estad MR Critical illness polyneuropathy: Risk factors and clinical consequences. A cohort study in septic patients Intensive Care Med 2001 27 1288 1296 10.1007/s001340101009 11511941

[B25] Gual P Gonzalez T Grémeaux T Barres R Le Marchand-Brustel Y Tanti JF Hyperosmotic stress inhibits insulin receptor substrate-1 function by distinct mechanisms in 3T3-L1 adipocytes J Biol Chem 2003 278 26550 26557 10.1074/jbc.M212273200 12730242

[B26] Zochodne DW Bolton CF Wells GA Gilbert JJ Hahn AF Brown JD Sibbald WA Critical illness polyneuropathy. A complication of sepsis and multiple organ failure Brain 1987 110 819 841 10.1093/brain/110.4.819 3651796

[B27] Goubel F Pigot A Allaf O Verleye M Gillardin JM Endotoxins modify muscle fatigue characteristics Fundam Clin Pharmacol 1995 9 202 204 10.1111/j.1472-8206.1995.tb00282.x 7628835

[B28] el-Dwairi Q Comtois A Guo Y Hussain SN Endotoxin-Induced skeletal muscle contractile dysfunction: Contribution of nitric oxide synthases Am J Physiol 1998 274 C770 C779 9530109 10.1152/ajpcell.1998.274.3.C770

[B29] Pereira LS Narciso FM Oliveira DM Coelho FM Souza Dda G Dias RC Correlation between manual muscle strength and interleukin-6 (IL-6) plasma levels in elderly community-dwelling women Arch Gerontol Geriatr 2009 48 313 316 10.1016/j.archger.2008.02.012 18462819

[B30] Frost RA Lang CH Skeletal muscle cytokines: Regulation by pathogen-associated molecules and catabolic hormones Curr Opin Clin Nutr Metab Care 2005 8 255 263 10.1097/01.mco.0000165003.16578.2d 15809527

[B31] Haddad F Zaldivar F Cooper DM Adams GR Il-6-Induced skeletal muscle atrophy J Appl Physiol 2005 98 911 917 10.1152/japplphysiol.01026.2004 15542570

[B32] Lang CH Pollard V Fan J Traber LD Traber DL Frost RA Gelato MC Prough DS Acute alterations in growth hormone-insulin-like growth factor axis in humans injected with endotoxin Am J Physiol 1997 273 R371 R378 9249574 10.1152/ajpregu.1997.273.1.R371

[B33] Frost RA Lang CH Differential effects of insulin-like growth factor I (IGF-I) and igf-binding protein-1 on protein metabolism in human skeletal muscle cells Endocrinology 1999 140 3962 3970 10.1210/en.140.9.3962 10465265

[B34] Bercker S Weber-Carstens S Deja M Grimm C Wolf S Behse F Busch T Falke KJ Kaisers U Critical illness polyneuropathy and myopathy in patients with acute respiratory distress syndrome Crit Care Med 2005 33 711 715 10.1097/01.CCM.0000157969.46388.A2 15818093

[B35] Sander HW Golden M Danon MJ Quadriplegic areflexic ICU illness: Selective thick filament loss and normal nerve histology Muscle Nerve 2002 26 499 505 10.1002/mus.10233 12362415

[B36] Amaya-Villar R Garnacho-Montero J Garcia-Garmendia JL Madrazo-Osuna J Garnacho-Montero MC Luque R Ortiz-Leyba C Steroid-induced myopathy in patients intubated due to exacerbation of chronic obstructive pulmonary disease Intensive Care Med 2005 31 157 161 10.1007/s00134-004-2509-9 15580474

[B37] Tang BM Craig JC Eslick GD Seppelt I McLean AS Use of corticosteroids in acute lung injury and acute respiratory distress syndrome: A systematic review and meta-analysis Crit Care Med 2009 37 1594 1603 10.1097/CCM.0b013e31819fb507 19325471

[B38] Fink H Helming M Unterbuchner C Lenz A Neff F Martyn JA Blobner M Systemic inflammatory response syndrome increases immobility-induced neuromuscular weakness Crit Care Med 2008 36 910 916 10.1097/CCM.0B013E3181659669 18431280

[B39] Deja M Hommel M Weber-Carstens S Moss M von Dossow V Sander M Pille C Spies C Evidence-Based therapy of severe acute respiratory distress syndrome: An algorithm-guided approach J Int Med Res 2008 36 211 221 18380929 10.1177/147323000803600201

