# Supplementary material for: Risk factors in critical illness myopathy during the early course of critical illness: a prospective observational study
Source: Crit Care. 2010 Jun 18;14(3):R119. doi: 10.1186/cc9074 (PMC2911767; doi:10.1186/cc9074)
Supplement: Additional file 1 — Further description of methods and definitions. The additional file contains additional information on exclusion criteria, electrophysiologic measurements, general ICU care, and laboratory testing [39]. Two tables within this file explain the conditions required for defining systemic inflammatory response syndrome, sepsis, severe sepsis, or septic shock (E1) and organ dysfunction (E2). [file cc9074-S1.DOCX]

Risk factors in Critical illness myopathy (CIM) during the early course of critical illness: a prospective observational study

Steffen Weber-Carstens MD^1(#)^, Maria Deja MD^1(#)^, Joachim Spranger PhD^2^, Florian Bubser MD^1^, Susanne Koch MD^1^, Klaus D Wernecke PhD^3^, Claudia D Spies PhD^1^, Simone Spuler PhD^4^, Didier Keh PhD^1^

(#) SW-C and MD are equally contributing first authors

# Online Data Supplement

## Methods

This study is a subanalysis of 40 patients derived from a prospective observational study (5). We excluded minors, patients with a history of preexisting muscle disorders, severe head trauma, or bleeding diathesis (thrombocytopenia < 20.000).

*Electrophysiological measurements:*

Electrophysiological measurements were performed with a Keypoint 2 portable electromyograph/neurograph (Medtronic, Skovlunde, Denmark). Myopathy according to abnormal dmCMAP after direct muscle stimulation was defined at dmCMAP values < 3 mV. Details of electrophysiological investigations are recorded elsewhere (5).

*Patients general ICU care:*

Patients were treated following standard operating procedures of intensive care (39) incorporating severe sepsis bundles (15). Systemic inflammation, sepsis or severe sepsis (Table E1, below) accompanied by organ dysfunction (Table E2, below) was classified according to consensus conference criteria (16,17). Patients were ventilated in a pressure controlled mode (biphasic positive airway pressure ventilation allowing assisted spontaneous breathing - BIPAP/ASB) to be aimed at a tidal volume of 6 ml/kg predicted body weight (PBW). Patients received standardised treatment including permissive hypercapnia, PEEP adjusted to oxygenation and intermittent prone positioning. Hemodynamic management followed early goal directed volume replacement as recommended by Dellinger et al. (15). Patients with septic shock received hydrocortisone with continuous infusion of 200 mg/day as an adjunctive therapy. Blood glucose adjustment followed a standardized protocol aiming at blood glucose levels <150 mg/dl. Neuromuscular blocking agents (NMBA) were used as bolus application only for specific maneuvers e.g. prone positioning or bronchoscopy.

Laboratory investigations:

EDTA blood samples were immediately centrifugated, and plasma was stored at -80 °C until batch processing. IL-6 and IL-10 was measured with ELISA (BD, Heidelberg, Germany) according to manufactures instructions. IGFBP-1 was measured with ELISA (Diagnsotics Systems Laboratories, Sinsheim, Germany). IGFBP-3 and IGF-1 were measured using the Immulite2000 (Siemens Healthcare Diagnostics GMBH, Eschborn, Germany). Quality measures were accurate respective all parameters (IGF-1: Intra-Assay CV 2.4-3.9%, Inter-Assay CV 3.7-8.1%; IGFBP-3: Intra-Assay CV 4.1-4.8%, Inter-Assay CV 5.2-7.3%).

**Table E1:** Definition of systemic inflammation, sepsis and severe sepsis

| SIRS |  |
| --- | --- |
|  | Two or more of: |
|  | Temperature >38°C or <36°C |
|  | Heart rate 90 bpm |
|  | Respiratory rate >20 1/min or paCO2 > 4.2kPa (32mmHg) |
|  | White blood cell count > 12 1/nl or < 4 1/nl or 10% immature (band) forms |
| Sepsis |  |
|  | SIRS associated with documented infection and at least one or more: |
|  | Altered mental state |
|  | Hypoxaemia: paO2 > 9.47kPa (72mmHg) at FiO2 21%, not due to a primary pulmonary disease |
|  | Elevated plasma lactate level |
|  | Oliguria (urine output < 0.5 ml/kg/h) |
| Severe sepsis |  |
|  | Sepsis associated with organ dysfunction, hypotension, or evidence of hypoperfusion, including but not limited to acutely altered mental status, oliguria, or lactic acidosis |
| Septic shock |  |
|  | Sepsis induced hypotension (systolic blood pressure < 90mmHg or mean arterial pressure < 60 mmHg or a fall of mean arterial pressure > 40 mmHg from baseline), not responsive to fluid resuscitation |

Criteria for the definition of SIRS, sepsis, severe sepsis or septic shock. SIRS: systemic inflammatory response syndrome, bpm: beats per minute; paCO2 partial pressure of carbon dioxide in blood: ;paO2 partial pressure of oxygen in blood: ;FiO2: fraction of inspired oxygen.

**Table E2:** Definition of organ dysfunction

| Organ dysfunction | criteria |
| --- | --- |
| Coagulation |  |
|  | 30% fall of platelets within 24h or platelets ≤ 100 000 /mm3, not due to acute hemorrhage or immunologic suppression |
| Lung dysfunction |  |
|  | Hypoxaemia: paO2 ≤ 10 kPa (75mmHg) at FiO2 21% or paO2-FiO2-ratio ≤ 33 kPa (250mmHg), not due to chronic disease of heart or lung |
| Renal dysfunction |  |
|  | Urine output < 0,5 ml/kg/h for at least 2h, not due to a hypovolemia or duplication of plasma creatinine above reference range |
| Liver dysfunction |  |
|  | Bilirubin > 2.0 mg/dl, without preexisting liver disease |
| Metabolic acidosis |  |
|  | Base excess ≤ -5mmol/l or lactate increase 1,5x above reference range |
| Mental State |  |
|  | Glasgow coma scale < 13 before start of analgosedation or |
|  | acutely altered mental status |

## Criteria for definition of organ dysfunction. paO2: partial pressure of oxygen in blood; FiO2: fraction of inspired oxygen.
